# COVID-19 Outbreak in an Amish Community — Ohio, May 2020

**DOI:** 10.15585/mmwr.mm6945a2

**Published:** 2020-11-13

**Authors:** Hammad Ali, Karthik Kondapally, Paran Pordell, Brandi Taylor, Gisela Medina Martinez, Ellen Salehi, Stacey Ramseyer, Susan Varnes, Nikki Hayes, Sietske de Fijter, Spencer Lloyd

**Affiliations:** ^1^CDC COVID-19 Response Team; ^2^Ohio Department of Health; ^3^Wayne County Health Department, Ohio.

In the United States, outbreaks of SARS-CoV-2, the virus that causes coronavirus disease 2019 (COVID-19), were initially reported in densely populated urban areas (*1*); however, outbreaks have since been reported in rural communities (*2*,*3*). Rural residents might be at higher risk for severe COVID-19–associated illness because, on average, they are older, have higher prevalences of underlying medical conditions, and have more limited access to health care services.* In May, after a cluster of seven COVID-19 cases was identified in a rural Ohio Amish community, access to testing was increased. Among 30 additional residents tested by real-time reverse transcription–polymerase chain reaction (RT-PCR; TaqPath COVID-19 Combo Kit),^†^ 23 (77%) received positive test results for SARS-CoV-2. Rapid and sustained transmission of SARS-CoV-2 was associated with multiple social gatherings. Informant interviews revealed that community members were concerned about having to follow critical mitigation strategies, including social distancing^§^ and mask wearing.^¶^ To help reduce the ongoing transmission risk in a community, state and county health department staff members and community leaders need to work together to develop, deliver, and promote culturally responsive health education messages to prevent SARS-CoV-2 transmission and ensure that access to testing services is timely and convenient. Understanding the dynamics of close-knit communities is crucial to reducing SARS-CoV-2 transmission.

## Investigation and Findings

On May 9 and May 11, 2020, respectively, a husband and wife in an Amish community in Wayne County, Ohio, experienced COVID-19–related symptoms. Both had nasopharyngeal samples tested and SARS-CoV-2 infection confirmed by receipt of positive RT-PCR results on May 14. The husband, who had a history of chronic obstructive pulmonary disease, participated in church services on May 2 and 3. He was hospitalized on May 15 with fever, cough, and shortness of breath, and received a diagnosis of COVID-19–related pneumonia; he was discharged on May 17. Another adult family member, with cancer, became symptomatic May 16, received a positive SARS-CoV-2 test result May 18, and died May 21. During May 13–19, four additional symptomatic community members received positive test results. After these initial seven cases were identified, community leaders contacted Wayne County Health Department (WCHD) to report that numerous other community members had symptoms** consistent with COVID-19. As a result, WCHD, with support from the Ohio Department of Health and a community bishop, organized a testing clinic at an Amish community school on May 20, where nasopharyngeal swabs were collected for RT-PCR testing. The testing clinic was publicized by the bishop and other community leaders; anyone could attend and receive testing.

CDC and Ohio health department investigators conducted 11 key informant interviews with community leaders and members. Some interviewees might have had COVID-19, but for reasons of confidentiality, interviewee names were not recorded. Consequently, interviews were not linked to cases. One interview was conducted at the testing clinic; 10 additional interviews, using snowball sampling (*4*), were conducted over the following 10 days. All invited participants orally consented to be interviewed. This activity was reviewed by CDC and was conducted consistent with applicable federal law and CDC policy.^††^ Interviews took 1 hour to complete and included open-ended questions to identify knowledge gaps related to COVID-19 prevention, transmission, and testing, and to understand attitudes, practices, facilitators of, and barriers to implementing strategies to decrease transmission. All interview notes were handwritten and reviewed by two interviewers. Theme saturation, a research term defined as the point “when a researcher begins to hear the same comments again and again” (*5*), was reached through iterative review and analysis. The following 10 themes were identified: 1) COVID-19 knowledge, including the spread of SARS-CoV-2; 2) myths and misinformation; 3) facilitators of and barriers to following COVID-19 prevention strategies at home, at work, and in the community; 4) use of traditional communication (e.g., newspapers) for information sharing; 5) access to testing; 6) means of transportation; 7) community cohesion; 8) selflessness; 9) strong work ethic; and 10) individual and community responsibility.

At the May 20 testing clinic and during the interviews, community members reported six social gatherings during the preceding 2 weeks, including a prechurch service^§§^ (May 2), church services (May 3, 10, and 17), a wedding (May 12), and a funeral (May 16) (Figure). Among 30 community members who had nasopharyngeal swabs collected at the testing clinic, 23 (77%) received positive SARS-CoV-2 test results. All community members with positive results reported multiple COVID-19–related signs and symptoms. The earliest symptom onset date was May 7, 5 days after a prechurch service and 4 days after a church service. On May 27, one person was hospitalized with fever and shortness of breath, received a diagnosis of COVID-19–associated pneumonia, and was discharged on May 30.

**FIGURE:**
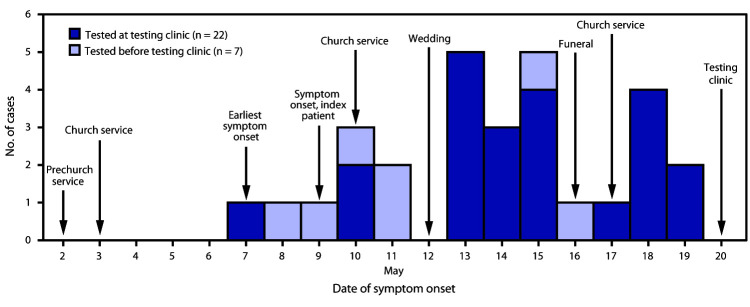
Date of symptom onset among 30* persons in an Amish community who received positive SARS-CoV-2 test results, dates of social gatherings in that community — Ohio, May 2–20, 2020 * Date of symptom onset missing for one patient tested at the testing clinic.

Among the 30 persons with laboratory-confirmed COVID-19, the mean age was 46 years (range = 12–86 years), and 21 (70%) were male. Eight of those persons reported having underlying medical conditions (Table). Symptoms most commonly reported included fatigue, headache, cough, myalgias, and chills. Among the 30 persons, none had traveled recently, and 24 (80%) at the time of testing reported contact with a person who was sick, usually at a social or religious event.

**TABLE:** Clinical characteristics of 30 persons with laboratory-confirmed COVID-19 in a rural Amish community — Ohio, May 2–20, 2020

Characteristic	No. (%)
Signs and symptoms	
Fatigue	24 (80)
Headache	21 (70)
Cough	17 (57)
Myalgias	17 (57)
Chills	16 (53)
Sore throat	15 (50)
Loss of taste or smell	14 (47)
Runny nose	12 (40)
Fever	11 (37)
Nausea or vomiting	9 (30)
Shortness of breath	7 (23)
Diarrhea	5 (17)
Underlying medical conditions	8 (27)
Cardiovascular disease/Hypertension	4 (13)
Diabetes	3 (10)
Immunocompromise	2 (7)
Chronic lung disease	1 (3)
Contact with a person with COVID-19 symptoms	24 (80)
Recent travel history	0 (—)
Hospitalized	3 (10)
Deaths	1 (3)

**Abbreviation:** COVID-19 = coronavirus disease 2019.

Most interviewees accurately reported knowledge about transmission and prevention measures, including that SARS-CoV-2 spreads through “coughing, sneezing” and can be prevented by “handwashing, social distancing, and staying at home.” However, several interviewees reported misconceptions that mask wearing might cause harm (“people wearing them all day long at work and getting a headache and not feeling well”), and that vitamins and herbs can help prevent SARS-CoV-2 infection. Several barriers to use of mitigation strategies were described, including having limited access to updated and trusted guidance (“access to health care is not an issue…access to good information is the problem”); lack of social or cultural acceptability of wearing masks (“the need to wear a mask has never been a part of this community”); and hesitancy around proper and consistent social distancing because of cultural practices and acceptability of the term (“fellowship is as important to us as worship,” “call it physical distancing…social distancing has the connotation of social isolation”). Interviewees also stressed the convenience and timing of testing clinics (“transport is a challenge because we need to hire a driver; testing clinic today made it easy” and “testing clinics should be coordinated with the communities”).

## Discussion

The Amish in Wayne County are part of the Greater Holmes County Area Settlement, which has the largest population of Amish in Ohio (36,955 in 2020).^¶¶^ Traditionally, the Amish limit engagement with the government, the non-Amish health care system, and modern medicine, except in acute events that affect the wider community, such as a 2014 measles outbreak in an Ohio Amish community (*6*), and prefer an herbal or natural approach to well-being (*7*).

The high SARS-CoV-2 positivity rates from the May 20 testing clinic and findings from the interviews highlighted the extent and probable reasons for community transmission and served to increase participants’ awareness of COVID-19. After the testing clinic, an additional 39 persons from the community received tests by June 28, after experiencing COVID-19–compatible symptoms or having close contact with a person with COVID-19. Among the 39 persons whose specimens were tested, 25 (67%) received positive test results, suggesting ongoing community transmission.

Amish communities emphasize strong social connections and communal activities (*7*). The importance of religious and social gatherings and communal fellowship among the Amish has challenged efforts to prevent infection during the COVID-19 pandemic. Six religious and social gatherings were reported in this community; such gatherings have been shown to lead to SARS-CoV-2 outbreaks (*8*). To help limit transmission within other Amish communities, public health officials recommended five strategies to local health departments. First, health departments should continue to build trusting relationships with Amish community institutions and leaders. Second, health education materials should be provided through local networks. The Amish rarely use electronic communication; however, well-established Amish media networks (newspapers and radio stations), local Amish steering committees (serving as liaisons to various government levels), and Amish- and non-Amish–owned businesses with Amish employees can help share COVID-19 prevention messages. Third, messages using culturally acceptable language emphasizing protection of family and community might help persuade community members to apply these strategies. Fourth, access to testing services needs to be timely and convenient, with active support from community leaders. Fifth, health departments and the community should continually share information and concerns about mitigation strategies and barriers to their use. Establishing points of contact within communities might allow health department staff members to promptly share updated or new information.

The findings in this report are subject to at least three limitations. First, the Amish community in which the outbreak occurred has diverse cultural practices and traditions. Some of the more traditional community members might have been reluctant to participate in the testing clinic, resulting in lower than expected turnout. Second, interviews were conducted from a convenience sample; therefore, findings might not be generalizable to this community or to other Amish communities. Finally, estimating COVID-19 attack rates among Amish communities is challenging. Amish communities are organized by church districts consisting of 20–40 families. Establishing the number of members in a specific community is difficult because members of one church district participate in other church districts’ religious and social gatherings, often based upon family ties.

Despite limited resources, strengthening collaboration between and across health departments and communities might help overcome cultural barriers. Although Amish communities might be experiencing challenges with preventing and mitigating SARS-CoV-2 transmission, leveraging Amish cultural beliefs of communal responsibility could help limit the spread of SARS-CoV-2.

**Summary**

**What is already known about this topic?**

COVID-19 cases have been increasing in rural U.S. communities. Social gatherings can facilitate exposure to and transmission of SARS-CoV-2.

**What is added by this report?**

Social gatherings, important in Amish communities, likely contributed to rapid transmission of SARS-CoV-2 in a rural Ohio Amish community. Some community members were concerned about having to follow critical mitigation strategies, including social distancing and mask wearing.

**What are the implications for public health practice?**

COVID-19 outbreaks in communities where social gatherings are common might be prevented by fostering collaborations and trust between the community and local health departments, sharing culturally and linguistically responsive health messages that emphasize protecting family and community members through established communication networks, and ensuring timely and convenient access to testing.

## Footnotes

* https://www.cdc.gov/coronavirus/2019-ncov/need-extra-precautions/other-at-risk-populations/rural-communities.html#why-higher-risk.

† https://www.fda.gov/media/136112/download.

§ https://www.cdc.gov/coronavirus/2019-ncov/prevent-getting-sick/social-distancing.html.

¶ https://www.cdc.gov/coronavirus/2019-ncov/prevent-getting-sick/diy-cloth-face-coverings.html.

** https://www.cdc.gov/coronavirus/2019-ncov/symptoms-testing/symptoms.html.

†† See e.g., 45 C.F.R. part 46.102(l)(2); 21 C.F.R. part 56; 42 U.S.C. ^§^241(d); 5 U.S.C. ^§^552a; 44 U.S.C. ^§^3501 et seq.

§§ Prechurch services are meetings of men of the church district to discuss the planning of upcoming services, including where and when it will be held, the message of the service, and needs of specific community members.

¶¶ https://groups.etown.edu/amishstudies/files/2020/10/Amish_Pop_by_state_and_county_2020.pdf.
